# Bioactive Coatings with Ag-Camphorimine Complexes to Prevent Surface Colonization by the Pathogenic Yeast *Candida albicans*

**DOI:** 10.3390/antibiotics10060638

**Published:** 2021-05-26

**Authors:** M. Joana F. Pinheiro, Joana P. Costa, Fernanda Marques, Nuno P. Mira, M. Fernanda N. N. Carvalho, Marta M. Alves

**Affiliations:** 1 Department of Bioengineering, Instituto de Bioengenharia e Biociências (iBB), Instituto Superior Técnico, Avenida Rovisco Pais, 1049-001 Lisboa, Portugal; mariajoana@live.com.pt; 2 Centro de Química Estrutural (CQE), Departamento de Engenharia Química, Instituto Superior Técnico, Universidade de Lisboa, Av. Rovisco Pais, 1049-001 Lisboa, Portugal; joanavcosta@tecnico.ulisboa.pt; 3 Centro de Ciências e Tecnologias Nucleares (C^2^TN), Departamento de Engenharia e Ciências Nucleares, Instituto Superior Técnico, Universidade de Lisboa, Estrada Nacional 10 (km 139,7), 2695-066 Bobadela LRS, Portugal; fmarujo@ctn.tecnico.ulisboa.pt

**Keywords:** Ag(I)-camphorimine complexes, *Candida* biofilms, bioactive coatings, antimicrobials and biocides

## Abstract

Currently there is a gap between the rate of new antifungal development and the emergence of resistance among *Candida* clinical strains, particularly threatened by the extreme adhesiveness of *C. albicans* to indwelling medical devices. Two silver camphorimine complexes, [Ag(OH){OC_10_H_14_N(C_6_H_4_)_2_NC_10_H_14_O}] (compound P) and [{Ag(OC_10_H_14_NC_6_H_4_CH_3_-p)}_2_(μ-O)] (compound Q), are herein demonstrated as having high inhibiting activity towards the growth of *Candida albicans* and *Candida glabrata* clinical strains resistant to azoles, the frontline antifungals used in clinical practice. Compounds P and Q were also explored as bioactive coatings to prevent colonization by *C. albicans* and colonize the surface of indwelling medical devices, resulting in persistent infections. Functionalization of stainless steel with polycaprolactone (PCL) matrix embedded with compounds P or Q was reported for the first time to inhibit the colonization of *C. albicans* by 82% and 75%, respectively. The coating of PCL loaded with Q or P did not cause cytotoxic effects in mammalian cells, demonstrating the biocompatibility of the explored approach. The identification and further exploration of new approaches for surface engineering based on new molecules that can sensitize resistant strains, as herein demonstrated for complexes P and Q, is a significant step forward to improve the successful treatment of candidiasis.

## 1. Introduction

Epidemiological surveys have shown that patients subjected to aggressive medical interventions, including the use of invasive indwelling medical devices (such as central venous catheters or pacemakers) or harboring implants, are at a higher risk of the development of invasive candidiasis, an infection that occurs when cells of the *Candida* genus disseminate through the bloodstream and may colonize any major internal organ [1]. This aggressive form of candidiasis has a mortality rate that can range between 30% and 60% (depending on the type of the infecting *Candida* species and on the patient’s underlying conditions) and is associated with prolonged hospital stays thus resulting in high morbidity [2]. Although it has been recognized the increase in the number of infections caused by non-*albicans Candida* species, in particular by *C. glabrata*, *C. albicans* is still responsible for more than 50% of the overall number of described cases of candidiasis [2,3]. The success of *Candida* species as pathogens, in special of *C. albicans*, is attributable, at least in part, to its remarkable ability to colonize biotic and abiotic surfaces, including all types of medical devices such as catheters, pacemakers, cochlear implants or prosthesis [4,5,6]. After the initial attachment and adhesion to the surface, *Candida* colonization progresses with the development of a thick biofilm that limits the activity of the host immune defense and drastically reduces the efficacy of antifungals [4,7,8,9]. Consequently, colonization of medical devices by *Candida* frequently requires their removal, often through surgery [10].

Numerous methods have been established to fight infections prompted by the colonization of medical surfaces by microbes, some of them applied to restrain colonization prompted by *Candida* species. One of the most successful approaches is by coating the surface with one (or more) molecules with recognized antimicrobial properties since this assures increased sterility of the material while preserving its bulk characteristics [11,12,13,14]. Polymeric coatings emerge in this context as particularly interesting considering the ability of polymers to form multivalent interactions to promote loading and, in some cases, also to allow the controlled release of drugs, including antifungals [6,15]. Although promising, two important obstacles to this approach are still recognized. One is the occurrence of eventual local and/or systemic toxic effects for the host cells caused by the presence of the drugs embedded in the coating and another is the persistent increase in the number of strains resistant to currently used antimicrobials [6,7]. Although far less advertised than resistance to antibiotics, the emergence of resistance to antifungals is very prominent and it has been increasing significantly in recent years [16]. To respond to this pressing challenge, several molecules showing anti-*Candida* activity have been identified [17,18], albeit in most cases the activity of these molecules has only been investigated in laboratory strains (often very different from clinical strains and with high susceptibility to antifungals), an important gap already identified in clinical methicillin-resistant *Staphylococcus aureus* strains [19]; no elucidation of the underlying inhibitory mechanism has been performed (which means that it is not possible to predict whether these molecules can inhibit growth of resistant strains); and it has not been disclosed whether the inhibitory effect results from the activity of the complex or from the ligand used in the synthesis process [6,7,20].

The present study is focused on the use of two silver camphorimine complexes, [Ag(OH){OC_10_H_14_N(C_6_H_4_)_2_NC_10_H_14_O}] (compound P) and [{Ag(OC_10_H_14_NC_6_H_4_CH_3_-*p*)}_2_(μ-O)] (compound Q), for which the synthesis had been previously reported [21,22]. The usefulness of camphorimine complexes as anti-*Candida* agents has been demonstrated in previous studies undertaken by our laboratories with the results showing that this activity can be determined by the structure of the complex and the steric and electronic characteristics of the camphorimine ligands [17,22]. Indeed, while the use of silver nitrate for the synthesis of camphorimine complexes resulted in complexes with a variable degree of activity against non-*albicans Candida* species, none of the synthesized complexes showed activity against *C. albicans* (presumably due to the capacity of this yeast to promote the formation of non-toxic Ag nanoparticles [17]), its replacement by silver acetate (the same precursor also used herein to synthesize P and Q) produced compounds with high activity against all *Candida* species, including against *C. albicans*. In our previous study [22], we could demonstrate that compound Q can inhibit the growth of the laboratory reference strains *C. glabrata* CBS138 and *C. albicans* SC5314; however, compound P was not studied. Prompted by these prior observations, in this study we have tested the ability of compounds P and Q to inhibit the growth of *C. glabrata* and *C. albicans* lab strains but also of a set of clinical strains resistant to fluconazole, the frontline antifungal therapy used in the clinical setting. The demonstrated efficacy of the two compounds in inhibiting the growth of *C. albicans* (including the tested azole-resistant strains) lead us to examine whether these molecules could be explored as bioactive coatings to prevent colonization prompted by these species. Poly(ε-caprolactone) (PCL), an FDA-approved polymer for use in tissue engineering and drug release systems [23,24] used with success for coating implants with antimicrobials [25,26,27], was selected as a matrix. The results obtained clearly demonstrate that the optimized strategy results in a significant reduction in the ability of *C. albicans* to colonize a stainless-steel surface without inducing any significant cytotoxic effects for mammalian cells, thus showing that the approach used can be interesting for surface engineering of medical devices, especially those more prone to be colonized by *Candida* cells.

## 2. Results and Discussion

### 2.1. The Camphorimine Complexes P and Q Inhibit the Growth of Azole-Susceptible and-Resistant C. albicans and C. glabrata Strains

Previously studied camphorimine complexes, and in particular, the compound Q used in this work ([{Ag(OC_10_H_14_NC_6_H_4_CH_3_-*p*)}_2_(μ-O)]), was described as an inhibitor of the growth of laboratory strains of *C. glabrata* and *C. albicans* [21]. This observation prompted us to examine whether compound P [Ag(OH){OC_10_H_14_N(C_6_H_4_)NC_10_H_14_O}] would also be inhibitory against the same pathogenic species. We included in the set of strains to be tested not only the lab strains *C. glabrata* CBS138 and *C. albicans* SC5314, but also a set of clinical strains that our laboratory has previously characterized as being resistant to fluconazole [17].

**Figure 1:**
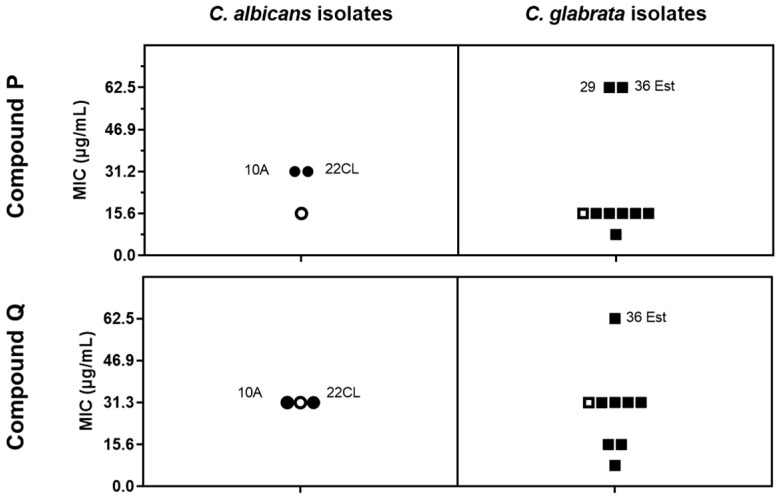
MIC values (µg/mL) of the complexes P and Q, obtained for laboratory strains *C. albicans* SC5314 and *C. glabrata* CBS138 (open symbols) and a set of azole-resistant clinical strains (closed symbols) (Table 1). The shown MICs represent the concentration of the compounds required to inhibit the growth of the strains in 50%, compared to the growth registered in a drug-free medium which was used as a control.

The results obtained depicted in Figure 1, show that against *C. albicans* the MICs of compound P ranged between 15.6 (obtained for the lab strain SC5314) and 31.2 mg/L (obtained for the two clinical isolates tested), while against *C. glabrata* the range was between 7.8 mg/L (obtained for one clinical strain) and 15.6 mg/L (obtained for the lab strain CBS138 and for remaining 6 clinical strains tested). For compound Q, the MIC against *C. albicans* was of 31.2 mg/L for the three strains examined, while against *C. glabrata* it ranged between 7.3 mg/L (obtained for one clinical strain) and 31.3 mg/L (obtained for the lab strain and for 5 clinical strains). There was no significant difference between susceptibility to P or Q of the azole-susceptible laboratory strains and of the azole-resistant clinical strains (Figure 1) thus suggesting that these compounds can sensitize the resistant strains.

**Figure 2:**
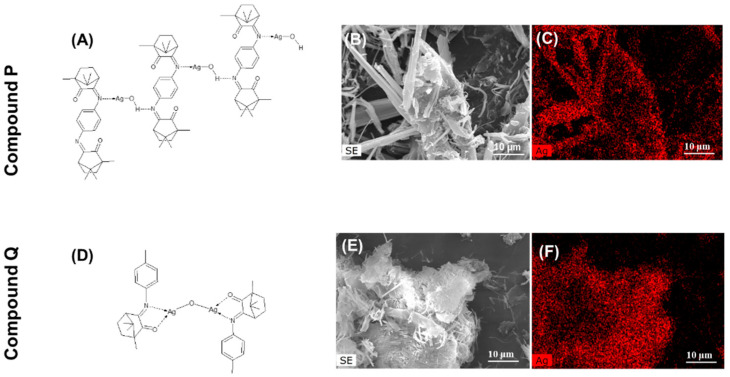
Physicochemical characterization of compounds P and Q; schematic structural arrangement (**A**,**D**), SEM micrographs (**B**,**E**), and EDS map of silver (**C**,**F**) of compound P and Q, respectively.

Despite the similarity in the antifungal activities of compounds P and Q, there are differences in the structure, since complex P fits in a coordination polymer arrangement (as detailed in Figure 2) while complex Q arranges as a binuclear complex (Figure 2C). Different morphologies were also observed for the two compounds, as revealed by scanning electron microscopy (SEM) micrographs (Supplementary Figure S1B,E) coupled with elemental composition analysis of Ag, N, O and C (detailed in SI, Figure S1A,B) obtained by X-ray energy dispersive spectrometry (EDS). Although compound P is formed by large needle-like structures (widths of ca. 2 µm) and micro-sized elongated blocks, compound Q shows small needle-like structures, with nano-sized widths and micro-sized round blocks. Despite the different morphologies, silver was detected in both compounds, as expected (Figure 2C,F). Our previous results also showed that the structure of camphorimine complexes can affect their inhibitory effect against microbial cells and, in particular, against *Candida* [21,22]; however, in the specific case of P and Q, this does not seem to impact their activity as the MICs were identical (Figure 1).

An attempt to elucidate the mechanism underlying the azole-resistance of *C. glabrata* strains used in this work revealed that they encode hyper-active variants of the transcriptional regulator CgPdr1 which, consequently, results in a potent up-regulation of the drug-efflux pump CgCdr1 [17]. The fact that the herein studied camphorimine complexes can sensitize these azole-resistant strains is an important feature since it might indicate that the efflux pumps over-expressed in these strains are not able to pump out the complexes potentiating their toxic effect. This observation is particularly important considering that the hyper-activation of CgCdr1, via CgPdr1 mutants, is the most common mechanism of resistance observed among *C. glabrata* azole-resistant strains [17]. It is also of remark the fact that the complexes were able to sensitize in a relatively identical manner *C. albicans* and *C. glabrata* cells, considering that cells of the latter species are known to be particularly resilient to environmental stress, including to antifungals [17].

### 2.2. Design of Bioactive Coatings with Silver Camphorimine Complexes

Considering the opportunist contamination of medical devices prompted by *Candida* species, especially by *C. albicans* which is the most relevant *Candida* colonizer of abiotic surfaces [28] and their relevance for the development of invasive infections, often life-threatening, and the above-demonstrated potential of compounds P and Q in inhibiting the growth of these species, we decided to examine whether these compounds could be used in bioactive coating of stainless steel (SS) surface to prevent colonization by the model yeast, *C. albicans*. Stainless steel was chosen as it is widely used in health care devices [28,29]. Bioactive coating by incorporation of compounds P and Q into the biodegradable polymer PCL was chosen, considered the prior use of this polymer for similar approaches [23]. Besides inhibiting the initial step of adhesion to the surface and, consequently, the formation of a biofilm, the envisaged bioactive coating may also promote the release of P and Q that may exert a toxic effect against planktonic cells.

One of the challenging steps in this quest is finding a suitable solvent to achieve PCL functionalization with P or Q without destroying the complexes. For that purpose, compound P and planktonic *C. albicans* cells were used to select the most convenient solvent for the coating formulation without affecting the physiology of the yeast cells. Three solvents, trifluoroacetic acid, dichloromethane and chloroform, exhibiting considerably distinct chemical properties (e.g., reactivity, miscibility, vapor pressure, etc.), as well as cytotoxicity, were tested (with or without additional DMSO) aiming at prompting PCL functionalized with P or Q.

**Figure 3:**
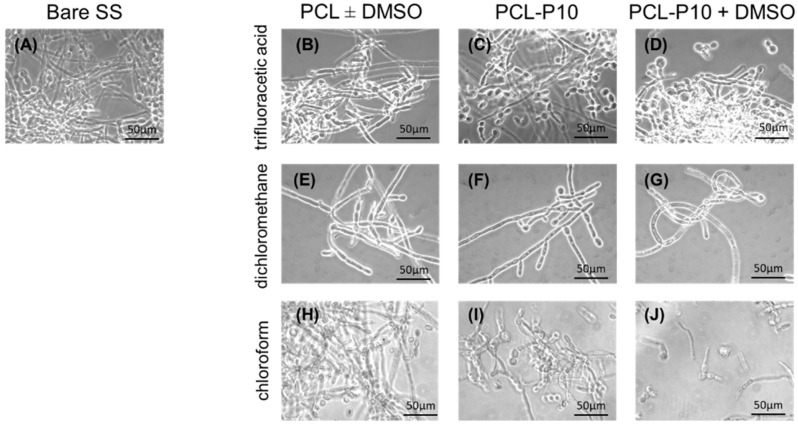
Analysis of planktonic *C. albicans* cells in the presence of different PCL coating formulations; representative optimal images of the planktonic cells observed by optical microscopy, with a magnification of 1000×, when in the presence of bare stainless steel (control, (**A**); PCL formulations using trifluoroacetic acid (**B**), dichloromethane (**E**) or chloroform (**H**), without or with DMSO; coatings only with PCL (**B**,**E**,**H**) or with embedded compound P (PCL-P10) without DMSO (**C**,**F**,**I**) or with DMSO (**D**,**G**,**J**).

As depicted in Figure 3A–D the use of trifluoracetic acid in the coating formulation resulted in normal growth of planktonic *C. albicans* cells (both in the yeast and hyphae form), as on the bare SS substrate (Figure 3A), indicating that trifluoracetic acid led to degradation of compound P and, consequently, did not inhibit yeast growth. When dichloromethane was used as a solvent, a clear reduction (Figure 3E–G) in planktonic yeast cells growth was observed in all the coatings, when compared with bare SS (Figure 3A); however, this was independent of the presence of compound P indicating that the observed inhibition resulted from a toxic effect of dichloromethane over the cells. When chloroform was used in the coating formulation (Figure 3H–J), the planktonic yeast cells preserved their growth and morphology, when compared with bare SS (Figure 3A), for PCL with or without DMSO (Figure 3H), showed a reduction when compound P was embedded in the coating in the absence of PCL (Figure 3I), and a dramatic morphological change and numerical decrease when DMSO was added to the formulation of PCL with the embed compound P (Figure 3J).

These results (Figure 3) were clear in showing that the best formulations for the bioactive coating with PCL with embedded compound P (i.e., planktonic yeast cells growth was preserved in the presence of PCL, but dramatically decrease in the presence of compound P), was obtained using a mixture of chloroform with 0.5% of DMSO; no altered chemical bonding of the polymer network was observed (SI, Figure S2A). A mixture of solvents, formerly reported as required for the successful formulation of a bioactive PCL coating with furanone [27], is in line with our observations that a combination of two solvents favored the coating bioactivity.

**Figure 4:**
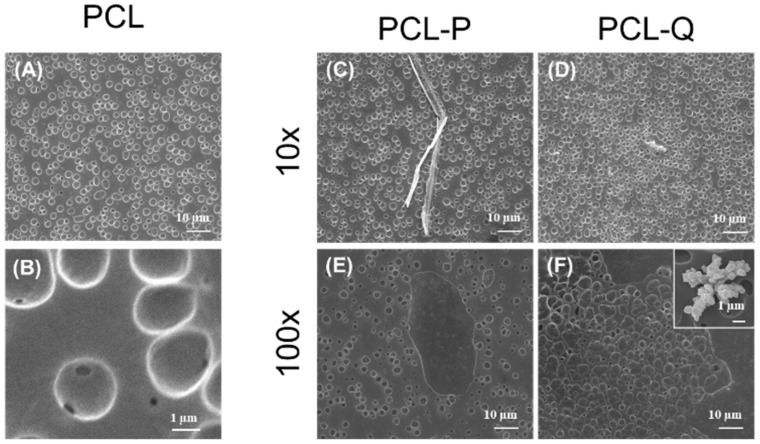
Physicochemical characterization of PCL coatings using chloroform in the coating formulation; SEM micrographs of the coatings with PCL alone (**A**,**B**) or with compounds P or Q: PCL-P10 (**C**), PCL-Q10 (**D**), PCL-P100 (**E**) and PCL-Q100 (**F**).

Two concentrations of P and Q, corresponding to 10 (P10 or Q10) or 100 (P100 or Q100) times the MIC obtained in the susceptibility assays undertaken, were used in the bioactive coatings’ formulation. The analysis of the physicochemical characteristics of these coatings showed that integrity was preserved and that the surface of the underneath substrate was fully covered, which are, of course, two necessary traits for successful bioactive coatings (Figure 4).

In the absence of complexes P or Q, the PCL coating obtained from solutions with DMSO displayed cavities (Figure 4A) not observed in typical PCL coatings formed in the absence of DMSO (SI, Figure S2B). The presence of DMSO alters the morphology of PCL in such a way that round cavities (2.2 ± 0.1 µm) evenly scattered on the surface are formed. Inside the cavities, the presence of small pores suggests that they can be interconnected by nanometric channel networks (Figure 4B). Similar morphology was depicted for PCL-P10 and PCL-Q10 coatings (Figure 4C,D) with cavities of 2.8 ± 0.2 and 2.5 ± 0.5 µm diameters, respectively. The typical morphologic features depicted by compounds P and Q (Figure 2) were found embedded in the polymeric matrix and the presence of Ag was corroborated by EDS (SI, Figure S1C,D). In the case of the bioactive coatings performed with the higher concentrations of P and Q (PCL-P100 and PCL-Q100) the morphologic characteristics were considerably different (Figure 4E,F) from those of PCL-P10 and PCL-Q10 (Figure 4C,D). In those coatings, cavities with micrometric (PCL-P100, 2.4 ± 0.7 µm, and PCL-Q100, 4 ± 1 µm) and nanometric cavities were observed. PCL-Q100 additionally displayed peculiar flower-like structures (inset in Figure 4F) that upon analysis by EDS shows to be predominantly composed of silver. Such data suggest that compound Q somehow rearranged during this coating formulation. Regardless of these differences in morphology, the results clearly show successful incorporation of Ag(I) camphorimine complexes P and Q in the PLC matrix without perturbing its integrity.

### 2.3. Effect of Bioactive Coating with P or Q in the Ability of C. albicans to Colonize Stainless Steel

**Figure 5:**
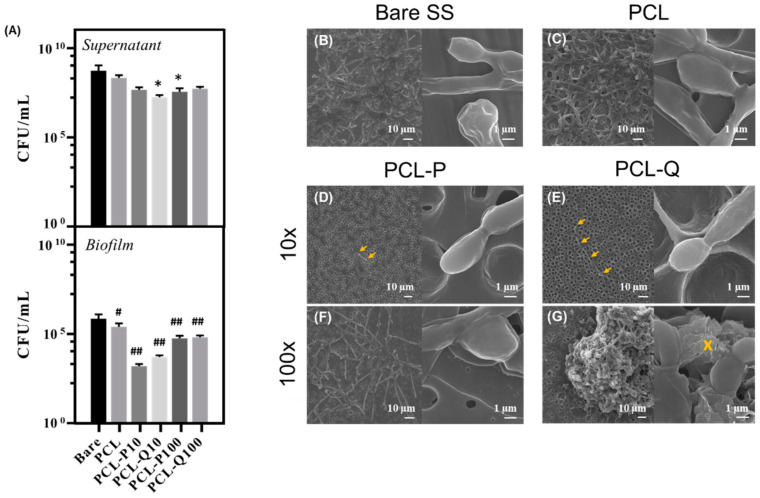
Colonization of bare SS, PCL and bioactive PCL coatings with P or Q compounds by *C. albicans* SC5314 incubated under simulated physiological conditions; graphical representation of CFUs/mL values obtained for each plate in the supernatant and biofilm (**A**), SEM micrographs of the bare SS plate (**B**), and PCL (**C**), PCL-P10 (**D**), PCL-Q10 (**E**), PCL-P100 (**F**) and PCL-Q100 (**G**) coatings, where the right hand-side SEM images are magnified yeast cells; Bars represent average errors and * or # *p*-values below 0.05 when compared with the corresponding control, while ## *p*-values below 0.001; Arrow marks point for isolated yeast cells; cross marks for particles predominantly composed by silver.

The results obtained so far allowed us to establish a protocol for optimized bioactive coatings on SS with compounds P and Q, and therefore we have decided to examine whether coatings with these molecules would reduce colonization prompted by *C. albicans* (Figure 5).

Coating with PCL alone resulted in a minor decrease in the ability of *C. albicans* to colonize SS surface. Similarly N, N-dodecyl, methyl-polyethyleneimine (DMPEI) coatings reveled antibiofilm activity [30]. When the PCL coating was loaded with P or Q, the number of cells adhered to the surface was very much smaller than the one recovered from bare SS plates, with P showing slightly higher efficacy than Q (Figure 5). Likewise, the functionalization of liposomal amphotericin B on polydimethylsiloxane (PDMS) prevented *C. albicans* colonization [31].

The increase in the amount of P in the coating increased its anti-*Candida* effect; however, for Q this resulted in an opposite effect (Figure 5). This observation can be attributable to the structural modifications of the PCL-loaded matrix with the higher concentration of Q that might decrease/modify the access of the compound to the *C. albicans* cells (Figure 4C,E respectively). SEM micrographs of the surface clearly show the impact that the presence of Q or P has in the colonization of the surface by *C. albicans* cells, since the thick biofilm that is observed on the bare SS surface (or in this same surface coated with PCL alone) is almost fully abolished when the surface is coated with PCL loaded with the camphorimine complexes (Figure 5). The presence of P or Q on the surface not only reduced the number of cells able to adhere to the SS surface, but also the formation of a biofilm since cells were scattered along the surface and not organized in multi-cellular structures, as demonstrated in the biofilms formed on the bare SS surfaces (Figure 5). This lower degree of colonization should result in the adhered *C. albicans* cells being much more prone to be removed from the surface, and therefore, increase their susceptibility to the action of antifungals and the host immune system. The increase in the amount of compounds P or Q in the PCL matrix did not improve antibiofilm capabilities and, in fact, it seemed to reduce it, since the number of *C. albicans* cells that we could recover from the surfaces was higher than the one obtained from surfaces coated with lower concentrations (Figure 5F,G). These results may suggest that the integrity of the silver complexes can be compromised when the ratio of PCL-to-complexes decreases. Although the PCL-P100 coating had a homogenous distribution of yeast cells on the surface (Figure 5F), the PCL-Q100 (Figure 5G) displayed the formation of yeast agglomerates around silver particles (where the camphorimine counterparts were no longer identified). These new silver-based structures act as growing spots and not as the desired antibiofilm compounds, corroborating that the reduction of Ag(I) to Ag(0) inhibits the antifungal activity of the Ag(I) camphorimine complexes [16,32]. A subtle, but detectable, reduction in the viability of *C. albicans* planktonic cells present in the supernatant where the coated SS surface was submerged suggests that some release of compounds P and Q could have occurred (Figure 5). This is, actually, in line with the demonstrated characteristics of PCL-loaded matrixes that can promote the controlled drug release [23].

### 2.4. Biocompatibility of the Coatings Functionalized with Silver Camphorimine Complexes

**Figure 6:**
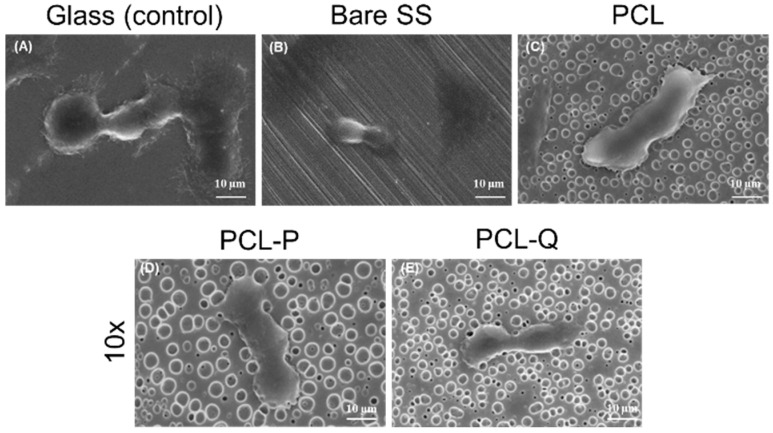
Characterization of the fibroblast colonizing the PCL coatings; SEM micrographs of fibroblasts colonizing the glass surface (**A**) that served as a positive control and of the plates, Bare SS (**B**); PCL (**C**); PCL-P10 (**D**) and PCL-Q10 (**E**) coatings.

To guarantee that PCL-P10 and PCL-Q10 are adequate coatings to be used in materials for implants, their cytotoxicity against mammalian cells was evaluated. Since fibroblasts are the most common cells of connective tissues, with a key role in their integrity, and have an active role in the maintenance of homeostatic mechanisms and response to pathophysiological conditions [33], these cells were used to assess the cytotoxicity of these bioactive coatings (Figure 6).

The use of fibroblasts cell cultures to access the cytotoxicity of materials intended to contact biological structures is outlined by the ISO guidelines [34]. Viability of 100% ensures that no toxicity arises from the bioactive coatings. To obtain a deeper insight into the biocompatibility of the coatings for mammalian cells, the integrity of the fibroblasts was investigated by SEM (Figure 6A–E). When comparing the morphology of the cells grown on the glass (Figure 6A) with those growing on the bare SS (Figure 6B), a clear spreading and typical round-spreading morphology is seen on the glass, although a reduced cell size is observed on the bare SS. The fibroblasts growing on the PCL (Figure 6C–E) or in the bioactive coatings with Ag-camphorimine complexes have morphologies such as the cells adhered on the glass (Figure 6A). Taking into consideration that compounds P and Q present considerable cytotoxicity for the V79 cells, respectively 12 and 8 µg/mL [17], the results suggest that the amount of compound released are below these values since no morphological alterations related to cell death were evident.

When evaluating the integrity of the coatings, the growth of both fungi or mammalian cells did not compromise PCL-P10 nor PCLQ10 structures, proving that these bioactive coatings are resistant enough to the required cell activity. Moreover, the cavities resulting from the interaction of DMSO with PCL (Figure 4A–C) is of special relevance. The resulting increase in surface roughness and porosity will favor the desired interaction with mammalians cells [35], whereas the subsequent surface increase will necessarily favor the antifungal and antibiofilm activities of these bioactive coatings.

## 3. Materials and Methods

### 3.1. Synthesis of Silver Camphorimine Complexes: Compounds P and Q

Silver camphor imine complexes [Ag(OH){OC_10_H_14_N(C_6_H_4_)_2_NC_10_H_14_O}] (compound P) and [{Ag(OC_10_H_14_NC_6_H_4_CH_3_-*p*}_2_(μ-O)] (compound Q) were synthesized from silver acetate by reaction with the suitable camphor imine ligands according to the reported procedures [21,22].

### 3.2. Strains and Growth Media

In this study, we made use of the laboratory reference strains *C. albicans* SC5314 and *C. glabrata* CBS138 and of 10 clinical strains, 2 from *C. albicans* (FFUL1 and FFUL2) [16] and 6 from *C. glabrata* (FFUL412, FFUL443, FFUL830, FFUL866 and FFUL874) [17] that were previously characterized as resistant to fluconazole (Table 1).

The strains were maintained at −80 °C in Yeast Peptone Dextrose (YPD) medium supplemented with 30% glycerol (*v/v*) (Merck, Germany). *Candida* cells were batch-cultured at 30 °C, with orbital stirring (250 rpm) in rich growth medium (YPD) or in RPMI (Roswell Park Memorial Institute Medium). YPD contains 20 g glucose *per* liter, (Merck Millipore), 10 g yeast extract (HiMedia Laboratories, Mumbai, India) and 20 g Peptone (HiMedia Laboratories). Cells were also cultivated in RPMI which contains, per liter, 20.8 g RPMI-1640 synthetic medium (Sigma, Germany), 36 g glucose (Merck Millipore, Germany), 0.3 g of L-glutamine (Sigma, Germany) and 0.165 mol/L of MOPS (3-(N-morpholino) propanesulfonic acid, Sigma, Germany). For the susceptibility assays, the pH of the RPMI growth medium was adjusted to 7.0 using NaOH (Sigma) as the alkalinizing agent. All media were prepared using deionized water. YPD medium was sterilized by autoclaving for 15 min at 121 °C and 1 atm while RPMI medium was sterilized by filtering the medium through a 0.22 μm pore size filter (Greiner bio-one Laboratories, Germany).

### 3.3. Assessment of C. albicans and C. glabrata Susceptibility to Compounds P and Q

Susceptibility of *C. albicans* SC5314, *C. glabrata* CBS138 and of the clinical strains to compounds P and Q was based on the determination of the minimum inhibitory concentration (MIC_50_), defined as the concentration that induces a growth reduction of, at least, 50%, compared to the growth observed in drug-free medium. The determination of MIC_50_ was performed in 96-multiwell polystyrene plates (Greiner bio-one Laboratories, Germany) and was based on the highly standardized microdilution method recommended by EUCAST (European Committee on Antimicrobial Susceptibility Testing) [36]. Briefly, *Candida* strains were cultivated at 30 °C with 250 rpm orbital agitation for 18 h in YPD growth medium and then diluted in 100 μL fresh RPMI growth medium (Sigma, Germany) to obtain a cell suspension with an OD_600nm_ of 0.05. These cell suspensions were mixed with 100 μL of fresh RPMI medium (control) or with 100 μL of RPMI medium supplemented with 0.98, 1.95, 3.91, 7.81, 15.63, 31.25, 62.5, 125.0, 250.0, 500.0 μg/mL of compounds P and Q. The stock solution of compounds P and Q were prepared using DMSO (dimethyl sulfoxide, Sigma, Germany) as a solvent. After inoculation, the 96-multiwell plates were incubated without stirring at 37 °C for 24 h. After that time, cells were resuspended and the OD_530nm_ of the cultures was measured using a microplate reader (SPECTROstar Nano, BMG LABTECH).

### 3.4. Bioactive Coating with Compounds P or Q Embedded in Polycaprolactone (PCL)

Stainless-steel (SS) plates were coated with polycaprolactone (PCL) embedded with compounds P or Q. Before coating, stainless-steel plates (316L SS, Goodfellow, UK) were polished with grit P600 and P1200 (Gravimeta, Spain) to ensure a homogeneous surface pattern, and then submerged in acetone (Sigma) to wash out any impurity. For the coating formulation, 4% (*w/v*) PCL pellets (Sigma, USA) were dissolved in trifluoroacetic acid (Sigma, Germany), chloroform (Carl Roth, Germany) or dichloromethane (Carl Roth, Germany), at room temperature, for 3 h using a magnetic stirrer. During this time two solutions of compounds P (0.156 μg/mL, PCL-P10 and 1.56 μg/mL, PCL-P100) and Q (0.313 μg/mL, PCL-Q10 and 3.13 μg/mL, PCL-Q100) were prepared using dimethyl sulfoxide (DMSO, Sigma, Germany) as a solvent; these values were chosen according to 10 × and 100 × the previously obtained MIC50 values. The obtained PCL solution was afterwards mixed with the prepared solutions of compounds P or Q, and the resulting solution used to coat the polished SS plates through dip-coating. The dipping process was made with a dip coater (RDC15), which controlled the removal of the plates from the PCL formulations at a speed of 0.3 cm s^−1^. After 1 min of drying, a second dip was made using the same conditions. The coated plates were allowed to dry overnight, in the dark, at room temperature (RT) and further analyzed (in terms of morphology and elemental chemical analysis) by scanning electron microscopy (SEM) (using a JEOL-JSM7001F or Hitachi S2400 apparatus) complemented with energy dispersive X-Ray spectrometer (EDS). To increase the conductivity of the samples, a conductive thin layer of gold and palladium was applied with a Polaron (E-5100).

### 3.5. Assessment of the Bioactive Coating in the Ability of Candida to Colonize Stainless Steel

The ability of *C. albicans* SC5314 to colonize bare SS surface or this same surface coated with PCL or with PCL loaded with P or Q, an experimental setting previously used with success was explored [37]. Briefly, *C. albicans* SC5314 cells were cultivated at 30 °C with 250 rpm orbital agitation in rich YPD growth medium until mid-exponential phase (OD_600nm_ = 1 ± 0.1) and then re-inoculated (at an initial OD_600nm_ of 0.1 ± 0.01) in a 25 mL capacity beaker containing 4.5 mL of fresh RPMI growth medium (Sigma, Germany). After inoculation, bare or coated SS plates were submerged in the prepared cell suspension and the system was incubated at 37 °C with gentle shaking (30 rpm) for 48 h. After that time, the plates were removed and washed twice with distilled water to eliminate non-adherent cells. The number of adherent viable cells was quantified upon scratching the biofilm formed on the surfaces and subsequently diluting the scratched material in 1 mL of sterile water. Serial dilutions were prepared (in a range of 10^−1^ to 10^−4^) and 50 µL of each cell suspension were plated on a solid YPD medium. The number of colony units (CFUs) formed after 48 h incubation, at 30 °C, was quantified. To estimate the number of CFUs present in the culture supernatant used to perform the colonization assays, 1 mL of each supernatant was diluted (1:10) in sterile water. Serial dilutions (in a range of 10^−1^ to 10^−5^) were prepared and 50 µL of each cell suspension plated on a solid YPD growth medium. To obtain further insights into the morphology of the biofilms formed on the surface of bare or coated surfaces by the different *Candida* strains the plates were analyzed by SEM. For this, colonized plates were washed with distilled water, immersed in 70% (*v*/*v*) ethanol (Sigma, Germany) for 10 min, transferred for a solution of ethanol 95% (*v*/*v*) ethanol and finally transferred again for absolute ethanol for 20 min to promote fixation. After completely air-drying, the plates were coated with a conductive thin layer of gold and palladium applied with a Polaron E-5100 and analyzed by SEM (using a JEOL-JSM7001F or Hitachi S2400 apparatus).

### 3.6. Cytotoxic Activity of the Bioactive Coatings

Normal fibroblasts (V79) (ATCC^®^ CCL-93, USA) were seeded in 6 well plates (2 × 10^5^ cells/2 mL) over bare SS, PCL and PCL loaded with compounds P or Q (PCL-P10 and PCL-Q10, respectively) in RPMI media supplemented with 10% FBS and 1% antibiotics (all from Invitrogen, USA). After 48 h incubation at 37 °C under a humidified atmosphere in a 5% CO_2_ incubator (Heraeus, Germany) the viability of the fibroblasts was quantified using trypan blue stain (Invitrogen) that exclusively labels dead cells. Quantification of the dead fibroblast cells under the different conditions tested was performed by light microscopy. As a control, the viability of fibroblasts cultivated in a glass was also performed.

## 4. Conclusions

Silver camphorimine complexes were already known to display high antifungal activities. The herein results represent a step forward since they show that complexes P and Q keep the antifungal activities while having suitable characteristics for the design of bioactive coatings. In fact, we succeeded in the formulation of coatings based on PCL and complexes P or Q, which upon biological assessment showed an ability to prevent surface colonization of stainless steel by *C. albicans*, while preserving the biocompatibility towards mammalian cells. The successful design of PCL-P and PCL-Q coating formulations is the first step in a process that requires further insights into parameters such as the anti-*Candida* activity on the surface properties, the effective amount of compound retained in the coating, the release profile as well as the coating thickness and adherence to the substrate. Additionally, properties such as the spatial orientation of the molecules in the polymeric matrix need clarification. Once these problems are met, it will be easier to develop drugs that have targets other than current drugs and design new compounds directed to new targets, for instance, clinical isolates. This is a field to explore since the complexes P and Q showed to be efficient against *C. albicans* (SC5314) and *C. glabrata* (CBS138) clinical isolates. Coatings based on compounds P or Q or newly designed/synthesized silver camphorimine complexes have a good perspective to cope with the growing resistance to antifungals and enlarge the panoply of available drugs.

To date, this is the first study demonstrating the potential of bioactive coatings with Ag(I) camphorimine complexes in preventing colonization by *C. albicans*. Achieving this inhibition is extremely important since biofilms are the mechanisms behind the success of this pathogenic yeast as a colonizer of medical devices. Increasingly in-depth research is essential for the design of more efficient indwelling and implant devices resistant to colonization by microorganisms capable of reaching clinical translation.

## Figures and Tables

**Table 1:** Reference and clinical strains used to test compounds P and Q.

Strains	
*C. albicans*	SC5314 (reference strain)
	10A
	22CL
*C. glabrata*	CBS138 (reference strain)
	FFUL29
	FFUL412
	FFUL443
	FFUL830
	FFUL866
	FFUL874
	8estef
	36estef

## Supplementary Materials

The following are available online at https://www.mdpi.com/article/10.3390/antibiotics10060638/s1, Figure S1: Chemical characterization of compounds P and Q, and PCL coatings; EDS spectra of compound P (A) and Q (B), and of PCL-P10 (C) and PCL-Q10 (D) coatings, Figure S2: Physicochemical characterization of PCL coatings using chloroform; Attenuated total reflectance-Fourier transform infrared (ATR-FTIR) spectra of PCL, PCL with dimethyl sulfoxide (DMSO) and PCL with DMSO and compound P (10 × MIC) (A); SEM micrographs of the PCL coating without DMSO in the coating formulation (B).
