# Supplementary material for: Bioactive Coatings with Ag-Camphorimine Complexes to Prevent Surface Colonization by the Pathogenic Yeast Candida albicans
Source: Antibiotics (Basel). 2021 May 26;10(6):638. doi: 10.3390/antibiotics10060638 (PMC8227220; doi:10.3390/antibiotics10060638)
Supplement: Supplementary file 1 [file antibiotics-10-00638-s001.zip › antibiotics-1205104-supplementary.pdf]

Bioactive coatings with Ag-camphorimine complexes to prevent surface colonization by the pathogenic yeast *Candida albicans*

M. Joana F. Pinheiro [1], Joana P. Costa [2], Fernanda Marques [3], Nuno P. Mira [1,*], M. Fernanda N. N. Carvalho [2,*], Marta M. Alves [2,*]

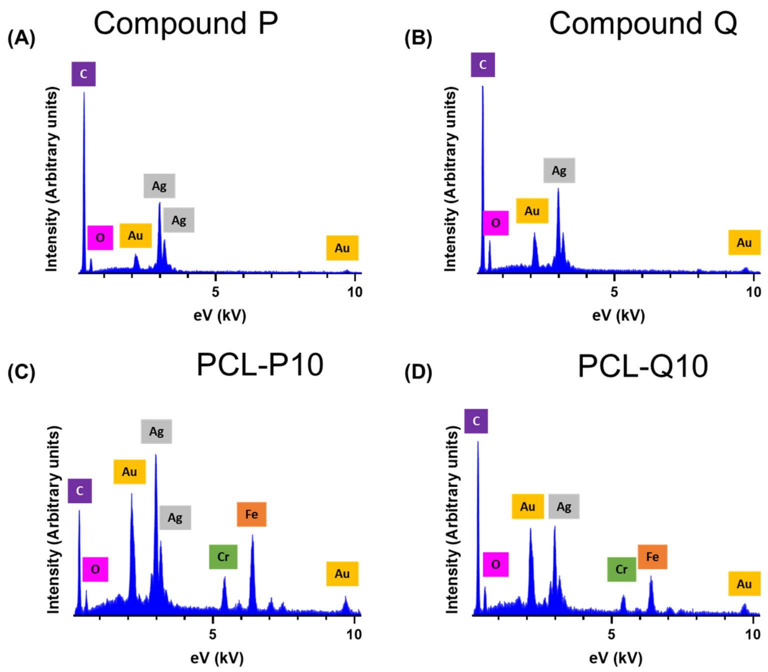

Figure S1 – Chemical characterization of compounds P and Q, and PCL coatings; EDS spectra of compound P (A) and Q (B), and of PCL-P10 (C) and PCL-Q10 (D) coatings.

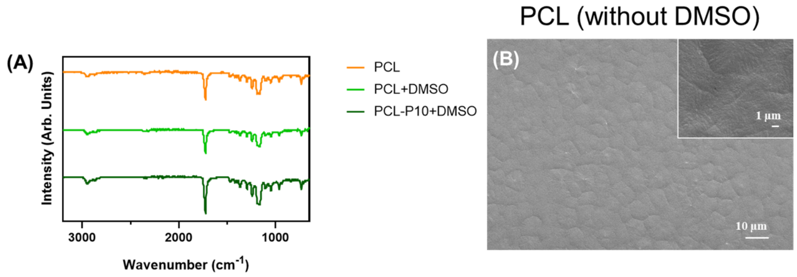

Figure S2 – Physicochemical characterization of PCL coatings using chloroform; Attenuated total reflectance-Fourier transform infrared (ATR-FTIR) spectra of PCL, PCL with dimethyl sulfoxide (DMSO) and PCL with DMSO and compound P (10x MIC) (A); SEM micrographs of the PCL coating without DMSO in the coating formulation (B).
